# Epigenetics of Host–Pathogen Interactions: The Road Ahead and the Road Behind

**DOI:** 10.1371/journal.ppat.1003007

**Published:** 2012-11-29

**Authors:** Elena Gómez-Díaz, Mireia Jordà, Miguel Angel Peinado, Ana Rivero

**Affiliations:** 1 Institut de Biologia Evolutiva (IBE, CSIC-UPF), Barcelona, Spain; 2 Institut de Medicina Predictiva i Personalitzada del Càncer (IMPPC), Badalona, Spain; 3 Maladies Infectieuses et Vecteurs: Écologie, Génétique, Évolution et Contrôle (MIVEGEC, UMR CNRS-UM2-UM1 5290, IRD 224), Centre IRD, Montpellier, France; International Centre for Genetic Engineering and Biotechnology, India

## Abstract

A growing body of evidence points towards epigenetic mechanisms being responsible for a wide range of biological phenomena, from the plasticity of plant growth and development to the nutritional control of caste determination in honeybees and the etiology of human disease (e.g., cancer). With the (partial) elucidation of the molecular basis of epigenetic variation and the heritability of certain of these changes, the field of evolutionary epigenetics is flourishing. Despite this, the role of epigenetics in shaping host–pathogen interactions has received comparatively little attention. Yet there is plenty of evidence supporting the implication of epigenetic mechanisms in the modulation of the biological interaction between hosts and pathogens. The phenotypic plasticity of many key parasite life-history traits appears to be under epigenetic control. Moreover, pathogen-induced effects in host phenotype may have transgenerational consequences, and the bases of these changes and their heritability probably have an epigenetic component. The significance of epigenetic modifications may, however, go beyond providing a mechanistic basis for host and pathogen plasticity. Epigenetic epidemiology has recently emerged as a promising area for future research on infectious diseases. In addition, the incorporation of epigenetic inheritance and epigenetic plasticity mechanisms to evolutionary models and empirical studies of host–pathogen interactions will provide new insights into the evolution and coevolution of these associations. Here, we review the evidence available for the role epigenetics on host–pathogen interactions, and the utility and versatility of the epigenetic technologies available that can be cross-applied to host–pathogen studies. We conclude with recommendations and directions for future research on the burgeoning field of epigenetics as applied to host–pathogen interactions.

## What Is Epigenetics?

**Figure 1:**
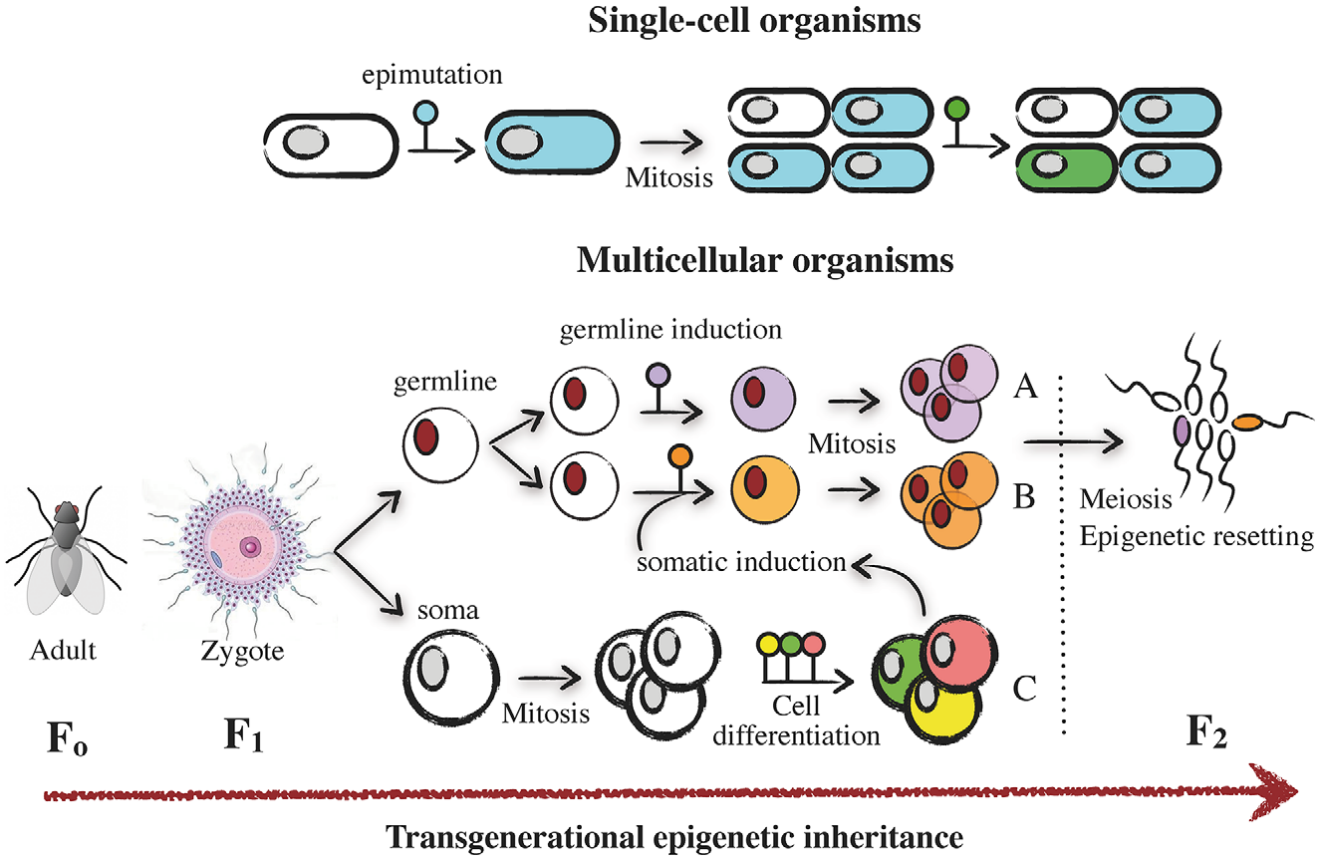
Mechanisms of epigenetic plasticity and inheritance. In single-cell organisms, epimutations induced by environmental stimuli (i.e., host) propagate in daughter cells by mitosis and result in transient or stable epigenetic states. In multicellular, sexually reproducing, organisms the zygote (F1) differentiates into germinal and somatic cells. Epimutations can be originated directly in the germline and propagated by mitosis (“germline induction”) (A), or they can arise and propagate as a consequence of interactions with the soma (“somatic induction”) (B). In the soma, after several rounds of cell divisions, epimutations tend to accumulate during cell and tissue differentiation processes (C). Only those epimutations generated in the germline that escape meiotic resetting during gametogenesis and oogenesis are expected to have transgenerational consequences (i.e., epigenetic inheritance) (F2).

Few areas in biology attract as much current attention and yet require as much presentation as the field of epigenetics. The term “epigenetics” was first used by Waddington to describe the process through which genotypes give rise to phenotypes during development [1]. Since then, there has been a burgeoning interest in the field of epigenetics that has been coupled with a diversification in the use of the term: epigenetics means different things to the different fields of biology, and even within a given field, different authors may use it in somewhat different contexts, generating a great deal of confusion in the process [2]. Broadly speaking, epigenetics refers to stimuli-triggered changes in gene expression due to processes that arise independent of changes in the underlying DNA sequence. Some of these processes have been elucidated and include DNA methylation [3], histone modifications and chromatin-remodeling proteins [4], and DNA silencing by noncoding RNAs (ncRNA) (BOX 1) [5]. This general definition of “epigenetics” is, however, used in two broadly different contexts. For some authors, the term “epigenetics” includes all transient changes in gene expression that occur at the individual cell level, as well as those that are propagated during mitosis in multicellular organisms and remain stable at the time scale of an individual (Figure 1). For clarity, we refer to this as *epigenetic plasticity* (see [6]). A good example is the development of morphologically different castes of bees from genetically identical individuals through nutritionally triggered DNA methylation [7]. Yet for other authors, and most notably for evolutionary biologists, the term epigenetics refers exclusively to *epigenetic inheritance*: the stimuli-triggered variation in gene expression that is heritable across generations. Here, the epigenetic changes are generated in the germ line in multicellular organisms (either directly or indirectly, see Jablonka and Raz [8]) or maintained clonally in single-cell organisms (Figure 1) [8], [9]. A classic example of transgenerational epigenetic inheritance involves a change in flower symmetry from bilateral to radial in *Linaria vulgaris*, which relates to different levels of methylation of the gene *Lcyc* [10]. In this review, we contend that both epigenetic plasticity and epigenetic inheritance are important in shaping host–pathogen interactions, and thus we use the term “epigenetics” to encompass both of these definitions.

**Box 1. The Epigenetic Code**

Specific combinations of epigenetic modifications constitute what has been called the epigenetic code, determining the functional (gene regulation, replication, repair, etc.) and structural features of each genomic region [68]. **Histone modifications**: A widely studied epigenetic mark is constituted by the set of *posttranslational modifications* (PTMs) on histones, which consist in the covalent addition of different chemical groups to particular residues, and that take place mostly in the tails of histones (see figure box). The association between different histone marks or variants and distinct chromatin and functional states (or *histone code* [69]) is well established. For instance, trimethylation of the histone 3 lysine 4 residue (H3K4me3) is usually linked to active genes, while trimethylation in lysine 9 residue (H3K9me3) is characteristic of repressed chromatin. **DNA methylation:** The DNA of most species is methylated and this modification takes place postreplicatively. In eukaryotes the modified base is 5-methylcytosine (5 mC) whereas in prokaryotes is mostly N6-methyladenine (6 mA) [70]. DNA methylation has a role in silencing gene expression and heterochromatin remodeling, among other functions [3]. DNA methylation patterns are dynamic and have changed several times through the tree of life, exhibiting a considerable structural, functional,and mechanistic diversity [71]. Hence, while in plants and vertebrates, DNA methylation occurs widely at CpG (C—phosphate—G) dinucleotide sites, regions of DNA where a cytosine nucleotide occurs next to a guanine nucleotide in the linear sequence of bases, and appear preferentially associated with transposons and silenced DNA; in invertebrates, DNA methylation is mainly found in gene bodies, but its regulatory function is only partially understood [72]. Interestingly, DNA methylation is not ubiquitous across the tree of life. Several species seem to have undergone loss of DNA methylation to a large degree, including model-species such as the nematode *C. elegans*, the insect *D. melanogaster*, and the yeast *S. cerevisiae*. **RNA-mediated silencing**: A variety of noncoding RNAs (ncRNA) have been shown to act in concert with the cell's epigenetic machinery, for example by establishing DNA methylation and by regulating histone modifiers [5]. Among those, the best characterized are the so-called microRNAs (miRNA), small ncRNAs of 19 to 24 nucleotides that bind target messenger RNAs and induce their translational repression, cleavage, or accelerated decay [73]. Yet the nature and function of this class of molecules are poorly understood, as well as the degree to which they contribute to epigenetic phenomena.

**Figure BOX 1:**
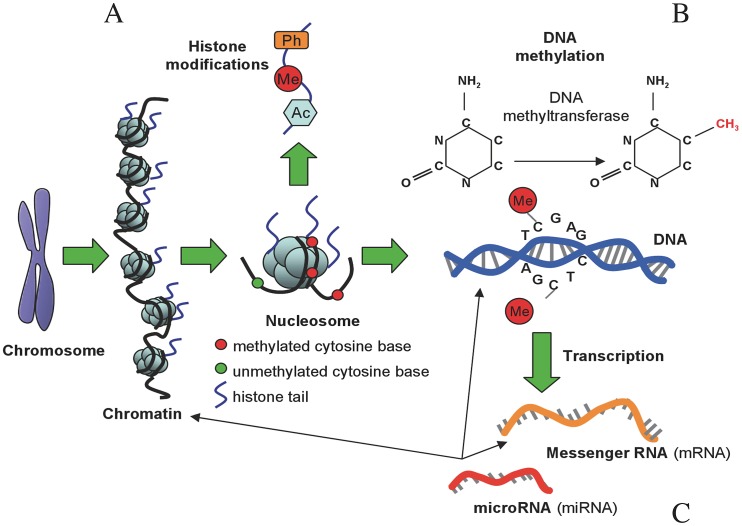
Types of epigenetic modifications. (A) Histones can undergo phosphorylation (Ph), methylation (Me), and acetylation (Ac), among other chemical modifications. These modifications are involved in chromatin remodeling and transcriptional regulation. (B) DNA molecules are methylated by the addition of a methyl group to carbon position 5 on cytosine bases, a reaction catalyzed by DNA methyltransferase enzymes, which maintains repressed gene activity. (C) mRNA is translated into a protein product, but this process can be repressed by binding of microRNAs (miRNA), a class of noncoding RNA (ncRNA). Figure adapted with permission from [45].

## Epigenetics of Host–Pathogen Interactions

**Figure 2:**
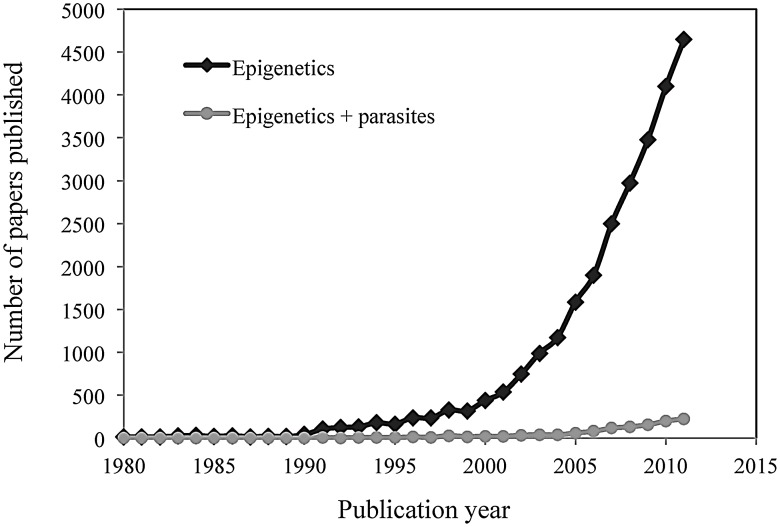
Comparison between the overall number of science citation-indexed publications in the field of epigenetics (black dots) and the number of such publications in the field of host–pathogen interactions (grey dots) over the last 30 years (1980 to 2011). Search carried out on the Web of Science (Thompson Reuters) on June 2012 using a date-restricted search (1980–2011) and “epigenet*” or “epigenet* and (parasite* or pathogen* or microbe* or bacter* or virus*)” as topic search terms.

In recent years, a plethora of papers on the role of epigenetic phenomena on gene expression and phenotype have brought about enormous progress in other fields (such as cancer epigenetics [11]) thanks in part to the significant advances of epigenetic technologies (see Box 2 and Table S1). Conversely, we still know comparatively little about the extent and significance of epigenetic variation in host–pathogen interactions (Figure 2).

**Figure 3:**
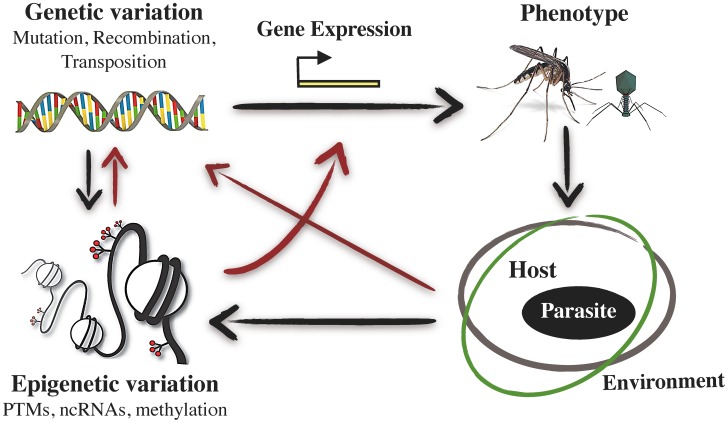
Schematic representation of the interrelations between epigenetic variation, phenotypic variation, and host–pathogen interactions. The infection phenotype, which varies between host and pathogen phenotypes and is environmentally dependent, can induce changes at both the genomic and epigenomic levels. These changes can in turn alter gene expression patterns. Apart from these direct effects of epigenetic variation on host and pathogen phenotypes, epigenetic variation can also have indirect, and transgenerational, phenotypic effects by influencing the probability of mutation, transposition, and/or recombination of the DNA sequence, as well as the predisposition of a gene with a particular epigenetic mark to be selected. See text for further explanation. Red arrows indicate action routes with potential inherited effects (see Figure 1).

**Box 2. Methods of Epigenetic Analysis**

Over the last decade, numerous techniques have been developed to analyze epigenetic marks at both genome-wide and sequence-specific levels. Here we summarize novel and cutting-edge methodologies that due to their versatile and straightforward nature can be cross-applied to host–parasite studies (refer to Table S1). A more comprehensive list of available technologies may be found elsewhere [11], [74].

*DNA methylation*

As a first step in any epigenetic study, **global DNA methylation analyses** allow the detection and identification of DNA methylation (either C and/or A methylnucleotides) and measure its frequency throughout the genome. These approaches do not require previous knowledge of the genome of reference, and most rely on a prior enzymatic/chemical hydrolysis of DNA to obtain the 2′-deoxymononucleosides, followed by the subsequent separation by chromatographic means such as *High Performance Liquid Chromatography* (HPLC) [75] or *High Performance Capillary Electrophoresis* (HPCE) [76], and a final detection step by UV spectroscopy or mass spectrometry. Alternatively, the global content of DNA methylation can also be quantified by enzymatic approaches such as the *Luminometric Methylation Assay* (LUMA) [77]. This technique is based on the digestion of DNA by methylation-sensitive and -insensitive isoschizomers (HpaII/MspI) and followed by pyrosequencing [78] to measure the extent of endonucleases cleavage.

Once the type of DNA methylation is determined, the next step is to study the **distribution and extent of DNA methylation**. The majority of methods are based on three strategies: DNA digestion by methylation-sensitive restriction enzymes, DNA bisulphite conversion, and affinity enrichment of methylated DNA using specific antibodies. The combination of these techniques with different molecular and analytical procedures has resulted in a plethora of approaches for determining DNA methylation patterns both at the specific and the genomic scales. At the scale of specific sequences, the **bisulphite sequencing** has become the gold-standard in mapping m5C sites at single base-pair resolution [79]. Following the bisulphite DNA treatment, cytosines in single-stranded DNA are deaminated to give uracil. After PCR amplification and DNA sequencing using primers that do not contain any CpG site, nonmethylated cytosines are recognized as thymines, while methylated cytosines remain as cytosines. This way, any cytosine that remains in bisulphite-treated DNA must have been methylated. But in recent years there have been major advances at the level of whole methylomes, and numerous techniques have been developed that now allow the study of DNA methylation at a genome-wide scale. The **Amplification of Inter-Methylated Sites** (AIMS) [80] is based on the differential enzymatic digestion of genomic DNA with methylation-sensitive and -insensitive isoschizomers (SmaI/XmaI) followed by the ligation of specific adapters and the amplification by PCR of the methylated sequences. Amplicons are resolved in denaturing polyacrylamide-sequencing gels, resulting in readable fingerprints that represent the organismal cell's DNA methylation profile. It has been widely applied to study DNA methylation in cancer [81], and more recently, to the discovery of DNA methylation in a social insect (*Apis mellifera*) [82]. Another straightforward approach is **Methylated DNA Immunoprecipitation** (MeDIP) [83], which is based on the isolation of methylated DNA fragments using an antibody specific for 5-methylcytosines. The utility of this technique depends upon the quality of the available antibodies, which at present limits the MeDIP analysis to 5 mC. Among the newest genome-wide technologies that can be applied to host–parasite studies, **microarray technology** provides a good resolution DNA methylation profiling, but its use is restricted to the availability of specific probes. In addition, in nonmodel organisms, a custom array must be designed. In recent years, fast advances in **Next Generation Sequencing** (NGS) have been successfully incorporated to analyze DNA methylation in a cost-effective manner, particularly when combined with enrichment techniques like the MeDIP-seq [84]. Nowadays, complete methylomes can be obtained at single-base resolution by sequencing bisulphite converted whole genomes [85]. This approach requires, however, complex bioinformatic analysis because bisulphite conversion significantly reduces the complexity of the genome by converting Cs into Ts, thus complicating the alignment of short reads to reference genomes. More recently, several new technologies under development that will reach the market during 2012 appear to be able to detect different DNA modifications directly without the bisulphite transformation. These technologies include the nanopore-based methods [86] and single molecule real time (SMRT) DNA sequencing [87].

*Modifications and variants of histones*

The identification and quantification of the *posttranslational modifications* (PTMs) and histone variants is an essential first characterization step, especially in nonorganisms models. **Mass spectrometry** is the gold standard in terms of accuracy [88]. However, most epigenetic research in this field focuses on detecting the association of individual proteins and histones with specific genomic regions. At present, the most powerful technique is **Chromatin ImmunoPrecipitation** (ChIP) [89]. After cross-linking DNA-binding proteins to DNA with formaldehyde in vivo, the chromatin is isolated and the DNA along with its associated proteins are sheared into small fragments. The DNA binding protein of interest is then precipitated using specific antibodies to isolate the complex, and as a final step, the cross-link is reverted to release the DNA. This method also relies on the availability and quality of antibodies. The immunoprecipitated DNA can be then analyzed by conventional or real-time PCR (ChIP-PCR) [90]. For genome-wide analyses, ChIP is followed by microarray hybridization (ChIP-on-chip) [91] or next-generation sequencing (ChIP-seq) [92]. ChIP-seq has become the state-of-the-art technology for mapping protein–DNA interactions in a genome-wide fashion, but data analysis is time-consuming and its application to nonmodel organisms is still limited.

Host–pathogen interactions are amongst the most plastic and dynamic systems in nature. To cope with the selective constraints imposed by their hosts, many pathogens have evolved an unparalleled level of phenotypic plasticity in their life history traits [12]. Likewise, the host phenotype is drastically and rapidly altered by the presence of a pathogen, and in some cases, the parasitized phenotype is inherited across host generations (see [13] for a review). In addition, co-adaptations between hosts and pathogens often occur over such short evolutionary time scales as to call into question the sole role of genetic modifications (i.e., mutation and/or recombination) as an underlying mechanism [14]. In this sense, epigenetic modifications may provide an accessory source of fast-acting, reversible, and readily available phenotypic variation that can be directly shaped by both host and pathogen selection pressures (Figure 3) [9], [14].

We describe herein recent examples of host–pathogen studies where epigenetic processes have already been shown to play a role and which can be broadly classified into (1) pathogen plasticity and (2) pathogen-induced alterations of the host (Table 1).

**Table 1:** Summary table of some of the best characterized epigenetic modifications (DNA methylation and histone posttranslational modifications [hPTM]) in host–pathogen interactions.

Topic/Organism	Epigenetic Mechanism	(E): Effectors, (T): Targets	Phenotype/Functions	Refs
**Parasite plasticity**				
*Plasmodium falciparum*	hPTM	**(E):** protein families ApiAP2, PfPuf2, PfGCN5, PfSET1, PfSET2, PfCARM1 & others **(T):** histones HP1, H3, H4, H2A, H2B, & others	Sexual & morphological differentiation (Transmission)	[15] [93] [22], [94] [18] [23], [24]
		**(E):** HACs & HMTs **(T)**: H3, Variant surface antigen families	Virulence (Antigenic variation)	
*Toxoplasma gondii*	hPTM & chromatin-modifying proteins	**(E):** HDAC, PRMT, MYST, GCN5, SET, ATP-dependent remodeling factors, **(T):** histones H3, H4, H2A, H2B	Sexual & morphological differentiation (Transmission)	[18], [20]
*Entamoeba histolytica*	hPTM	**(E):** *nd*, **(T):** Histones H3, H3K4, *ap-a*, *cpA5* & *lgl1* genes	Virulence (Cytotoxicity)	[25]
*Salmonella enterica*, *Escherichia coli*, and others	DNA adenine methylation	**(E):** CcrM and DAM, **(T):** pathogenicity island I (SPI-1), *lppB* gene, *std* & *spv* operon	Virulence (Motility, Cell adhesion & Invasion, Cytotoxicity)	[28]
*Candida albicans*	DNA methylation	**(E):** *nd*, **(T):** RPD3, PBI2, FOX2 genes	Morphological differentiation (Transmission)	[21]
*Giardia lamblia*	hPTM	**(E):** HAC & HDAC, **(T):** *nd*	Morphological differentiation (Transmission)	[95]
*Schistosoma mansoni*	DNA methylation	**(E):** DNMT2, **(T):** Smp155010 protein	Development (Oviposition)	[96]
*Epstein-Barr Virus (EBV)* and others	DNA methylation	**(E):** Human cellular DNMTs, **(T):** Viral methylome	Virulence	[97]
**Pathogen-induced host alterations**				
*Wolbachia pipientis*	DNA methylation	**(E):** prophage DNMTs?, **(T):** *nd*	Male feminization	[39], [40]
*Pseudomonas syringae*	hPTM & DNA methylation	**(E):** HAC & HMT, **(T):** WRKY6 & WRKY53 genes; DEFENSIN1.2 promoter	Host immune priming	[44]
*Influenza virus*	hPTM & DNA methylation	**(E):** Viral NS1 (ARSK sequence), **(T):** transcription factor PAF1	Host immunosuppression	[37]
*Human adenovirus (HAdV)*	hPTM	**(E):** Viral E1A protein **(T):** IFN signaling, Histone H2B	Host immunosuppression	[98]
*Mycobacterium tuberculosis*	hPTM & chromatin-modifying proteins	**(E):** SWI/SNF protein complex **(T):** IFN signaling, CIITA promoter	Host immunosuppression	[36]
*Listeria monocytogenes*	hPTM & chromatin-modifying proteins	**(E):** Listeriolysin O, **(T):** MAPK signaling, Histone H4 and H3	Host immunosuppression	[33]
*Toxoplasma gondii*	hPTM & chromatin-modifying proteins	**(E):** *nd*, **(T):** IFN signaling, transcription factor STAT1	Host immunosuppression	[99]
*Anaplasma phagocytophilum*	hPTM & chromatin-modifying proteins	**(E):** HDAC1, **(T):** Histone H3	Host immunosuppression	[100]

IFN, Interferon; MAPK, mitogen-activated protein kinase; HAC/HDAC, Histone acetylase/deacetylase; HMTs, Histone methyltransferase; DAM, DNA Adenine Methyltransferase; DNMT, DNA methyltransferase.

## Pathogen Plasticity

One of the most notorious aspects of pathogens is the morphological and developmental plasticity they exhibit, which is intimately linked to their survival and transmission in the host. Complex life-history transitions that occur in response to the changing host environment require rapid and profound alterations of their gene expression profiles. Take, for example, the malaria parasites in the genus *Plasmodium*. In the vertebrate host, the parasite has distinct hepatocytic and erythrocytic stages, and it forms sexually differentiated gametocytes in the blood that are taken up by the mosquito, where these gametocytes mate, then migrate through the midgut to form oocysts and from there to the salivary glands as sporozoites. Previous studies have revealed distinct gene expression profiles in all of these phases (reviewed in [15]), suggesting that developmental switches are transcriptionally regulated. However, apicomplexan parasites such as *Plasmodium* are notoriously poor in transcription factors [16]. In contrast, these parasites contain a rich repertoire of histone variants, chromatin and histone modifying enzymes, and RNA-mediated silencing mechanisms [17], [18]. In *Toxoplasma gondii*, histone acetylation has been shown to be responsible for the switch between the replicative and nonreplicative stages of the pathogen [19], [20]. Similar mechanisms of epigenetic regulation have been characterized in other protists (Table 1). Although less studied, *Trypanosoma brucei* is the only Apicomplexa where DNA methylation has been detected, but the significance of these epigenetic modifications in parasite cell-cycle regulation remains unexplored [18]. More recently, DNA methylation has been shown to be responsible for the transition between the yeast and hyphal forms of the polymorphic yeast *Candida albicans* [21].

The second striking aspect of pathogen plasticity concerns their ability to alter the expression of genes linked to virulence processes, which allows them to colonize, replicate, and/or disseminate within the host. Within the Apicomplexa, *Plasmodium falciparum* switches its variant surface proteins during its erythrocytic stage to avoid the host's immune system (antigenic variation). These surface proteins are encoded by highly polymorphic gene families (*var*, *rif*, *stevor*, and *pfmc-2tm*, among others). In the case of the *var* family, the ability of the parasite to express only one of the 60 genes that encode for these proteins (called PfEMP1) is epigenetically regulated through histone modifications [22]. In a very recent study, Rovira-Graells et al. [23] have reported a more general association between these histone and chromatin marks and clonally variant expression, extending previous results on *Plasmodium var* genes to all but two of the 28 variantly expressed gene families. In addition, recent work has shown that the epigenetic state of the parasite is maintained during several rounds of cell division [24]. Epigenetic control of virulence factors is well demonstrated in several microbial pathogens. In *Entamoeba histolitica*, for example, histone methylation and demethylation regulate the expression of the amoebapore protein (a protein responsible for the cytotoxicity of the pathogen [25]). DNA methylation is also an essential regulatory mechanism of virulence in several pathogenic bacteria [26], [27]. In *Salmonella enterica*, for example, lack of *Dam* (DNA adenine methyltransferase) methylation causes, amongst other things, envelope instability, reduced motility, and an impaired ability to invade the intestinal epithelium [28].

Given the importance of epigenetics for pathogen biology, understanding how the host environment cues the epigenetic transition between the replicative and transmission stages and the virulence factors of morbid and deadly parasites such as *Plasmodium* is not only an academic exercise, but it will also provide novel targets for drug development; an option that has been termed “*epigenetic therapy*” is currently being tested in clinical trials for other (noninfectious) diseases. These prevention and treatment strategies translated to the field of host–parasite interactions could be aimed at arresting the developmental switches of parasites within the host or at blocking or limiting their virulence. This could be achieved by using chemical inhibitors, gene knockout, and RNA interference (RNAi) approaches, designed to target the epigenetic machinery of the parasite such as the DNA methyltransferases or the chromatin and histone modifying enzymes (see Table 1, [29]).

## Pathogen-Induced Alterations of the Host

Pathogen-induced alterations of host physiology, morphology, and behavior are widely documented in the scientific literature. Perhaps the most fascinating examples of these changes are those that have been shown to be the result of a manipulative strategy of the pathogen aimed at maximizing its survival and transmission. Although some of the mechanisms underlying such pathogen manipulation have been unraveled [30]–[32], by and large, we know startlingly little of the strategies used by pathogens to achieve this end. In the last few years, however, evidence has accumulated that histone modifications and chromatin remodeling regulate gene expression and are thus key targets for pathogen manipulation during an infection [33]. One such obvious target is the host's immune system. In recent years, the epigenetic modulation of host's transcriptional program linked to host defense genes has emerged as a relatively common occurrence of pathogenic viral and bacterial infections [33], [34]. Bacteria are the hallmark of epigenetic studies on microbes and provide several pioneer examples on infection-induced host gene reprogramming [32]. A diverse array of bacterial effectors has been identified that either mimic or inhibit the host cellular machinery, thus facilitating the pathogen's life-cycle. MAPK (mitogen-activated protein kinase), Interferon (IFN), and transcription factor NF-κB signaling pathways are common targets of bacterial-induced post-translational modifications, acetylation, ubiquitylation, and phosphorylation on histones and chromatin-associated proteins [35]. Within the alveolar macrophages, *Mycobacterium tuberculosis*, for example, inhibits interferon-γ-induced expression of several immune genes through histone acetylation [36], which explains the persistence of long-term chronic tuberculosis infections in some patients. This mechanism is not restricted to bacteria but appears rather ubiquitous among intracellular pathogens (Table 1). Influenza viruses go a step further at circumventing host immune defenses. In a recent study, Marazzi et al. [37] report an influenza protein called NS1 that contains an amino-acid sequence (ARTK) very similar to the host's H3 histone tail. The authors provide compelling evidence of how using this histone mimic sequence, the viral NS1 protein hijacks a host transcription elongation factor (hPAF1), selectively suppressing the cell's production of antiviral proteins. This work is a good example of how a molecule of pathogen origin can directly induce an epigenetic modification in the host. More studies are needed that establish causative relationships between the pathogen infection and host epigenetic modifications such as DNA methylation and posttranslational histone modifications. Indeed, most of the evidence currently available is correlational (but see Table 1), and cases where proteins of pathogen origin have been shown to interact directly with the host epigenetic machinery are still scarce.

An additional characteristic of many pathogens is their ability to manipulate the reproductive biology of their hosts. The endobacteria *Wolbachia pipientis* is the archetypal example of such reproductive manipulations. *Wolbachia* is the most common parasitic microorganism in insects. Its maternal inheritance has selected for a variety of phenotypes associated with manipulating the reproduction of its hosts: forcing asexuality, feminizing hosts, killing males, and inducing incompatibility between infected males and uninfected (or differently infected) females [38]. Negri et al. [39] have provided the first evidence that a feminizing strain of *Wolbachia* interferes with the genetic imprinting of its host (the leafhopper *Zyginidia pullula*) by altering the host's methylation pattern. Recently, the widespread existence of putative DNA-methyltransferases in the prophage of the *Wolbachia* infecting several *Drosophila* species [40] has raised the possibility that this may be a widespread mechanism of epigenetic interference in this endosymbiotic bacteria. The link is, however, unclear since these enzymes have been identified as adenine methyltransferases, a family of prokaryotic enzymes that methylate the amino group at the C-6 position of adenines, whereas in the example reported above, genetic imprinting of the invertebrate host seems to occur at the C-5 carbon of CpG cytosines.

Not all modifications that take place in the infected host are, however, adaptive for the pathogen. Some of them are adaptive strategies of the host aimed to compensate or minimize the effects of the infection. In vertebrates, invertebrates, and plants, individuals that have recovered from certain infectious diseases are protected against later infection with those same diseases (immune priming). While the mechanistic basis of immune priming in invertebrates is still unresolved, in vertebrates, histone modifications may be associated with immune memory following a viral infection in CD8 T cells (reviewed in [41]). Histone modifications, DNA methylation, and other chromatin remodeling mechanisms, including deposition of histone variants and ATP-dependent chromatin remodelers, also seem to serve as a memory for priming in plant immunity [42], [43]. In some cases, acquired immunity can be passed from mother to offspring, endowing the offspring with improved defense against infection (transgenerational immune priming). For instance, a very recent paper has shown that in *Arabidopsis thaliana*, immune priming to *Pseudomonas syringae* is transmitted between plant generations through the hypomethylation of defense-related genes [44].

## Future Directions

In this review, we have concentrated our attention on the current evidence available for the role of epigenetic mechanisms in pathogens' life cycle and pathogen-induced modification of host phenotype. However, epigenetics not only represents a paradigm shift in our understanding of host and pathogen phenotypic plasticity. We believe that in the next few years, perhaps the most exciting developments in the field of epigenetics will come by linking epigenetic variation and inheritance to the epidemiology and evolution of infectious diseases.

Epigenetic epidemiology has recently emerged as a promising area for future research on infectious diseases [29], [45]. In recent years, disease association studies based on epigenomic mapping have arisen as a powerful tool for disease risk prediction in humans. But these studies typically face the “chicken-and-egg” causality problem: there is an association between a particular disease phenotype and the epigenome, but it is not easy to establish whether it is the disease which is causing the epigenetic changes or whether the epigenetic changes are the ones causing the disease pathogenesis [29]. New epidemiological approaches are, however, being developed in epigenetic disease studies to control for such cause–effect relationships [46]. However, the reversible and context-dependent nature of epigenetic changes poses serious caveats to epidemiological studies. For example, many epigenetic changes linked to disease risk can be lost after one generation, change from tissue to tissue, or be differentially expressed in an age-dependent, sex-, and parent-of-origin-specific manner [47]–[49]. To overcome these difficulties, epigenetic studies of disease must be accompanied by comprehensive longitudinal (multistage and multi-individual) and transgenerational data. Although there is a lot of effort to bring epigenetics into epidemiological research in several noninfectious human diseases (see examples reviewed by [50], [51]), we still know very little about the consequences of epigenetic processes in the emergence and epidemiology of infectious diseases. Therefore, a comprehensive survey of epigenetic determinants of pathogenesis coupled with population-level epigenetic diversity studies in host–pathogen systems is needed before any disease prediction and prevention strategies can become a reality [52].

The second area of research is the role of epigenetic variation in *host and pathogen coevolution and evolution*. Since the incorporation of epigenetic inheritance and epigenetic plasticity mechanisms to evolutionary models and empirical studies of host–pathogen interactions is still lacking, our discussion is necessarily speculative. However, we envisage several areas that are ripe for future research. Models of host–pathogen co-evolution assume the presence of genetic variation for host resistance and pathogen infectivity, as well as genotype-specific interactions [53]. Mechanisms of genetic variation alone are, however, often unsatisfactory to explain the compatibility between host and pathogen phenotypes [54], and nongenetic inheritance mechanisms may have an important role to play (e.g., [55]–[57]). In addition, host–pathogen co-evolutionary interactions are often context-dependent (i.e., spatially and temporally variable), and the output of infection often depends upon several environmental factors such as temperature or nutrition [58]. Given the prominent role of epigenetic processes in environmentally induced phenotypic plasticity and adaptation [59], the fact that genotype-by-environment interactions in host–pathogen systems are epigenetically regulated seems a reasonable assumption. The work by Laine et al. [60] on a fungal pathogen and its host plant has, for example, demonstrated a temperature-dependent effect on pathogen performance on local versus foreign hosts. Multiple cases of environmentally triggered co-adaptations have been reported in several other host–pathogen systems (reviewed by [61]), which we contend will provide the raw material for future epigenetic studies.

A further unresolved matter is to establish the extent, nature, and significance of *epigenetic inheritance* in host–pathogen interactions. For example, transgenerational immune priming in invertebrates [62]–[64] and plants [42] is likely to have an epigenetic component, but the actual mechanism of inheritance is not known. Other transgenerational infection effects on host behavior and physiology, often so-called maternal effects, still need to be investigated (reviewed by [13]). Several authors (Bonduariansky and Day [9], Bossdorf et al. [65], Ho and Burggren [66]) have provided recommendations for testing epigenetic inheritance experimentally, which could be cross-applied to host–pathogen studies (Figure S1). In addition, there is an urgent need for epigenetic studies to develop solid theoretical evolutionary models [67]. Bonduriansky and Day [9] have suggested that epigenetic inheritance allows us to overcome three major limitations of genetic inheritance on phenotypic evolution: (1) It allows for traits acquired during the lifetime of an individual to be directly transmitted to the offspring, (2) it allows the transmission of favorable trait combinations across generations (genetic recombination tends to break such combinations), and (3) it provides an additional source of phenotypic variation for selection to act upon. At present, however, we know startlingly little about how these phenomena may impact the evolution of host–pathogen interactions. In this sense, the collaboration between molecular epigeneticists, functional and experimental parasitologists, and theoretical evolutionary biologists is needed to extend the current gene-based view of host–pathogen interactions into a more integrated one that includes both genetic and epigenetic dimensions.

## Concluding Remarks

In recent years there has been an explosion in the number of epigenetics papers across biological disciplines (Figure 2), a progression that has been accompanied by technological breakthroughs that now make it possible to undertake sophisticated epigenomic studies across a range of organisms (Box 2 and Table S1). However, studies on the complex and multifaceted co-evolutionary interactions between hosts and pathogens have received comparatively little attention (Figure 2), and this in spite of their potentially evolutionary and epidemiological implications.

In this review, we have concentrated our attention on the current evidence available for those few cases in which an epigenetic mechanism has been described (i.e., see Table 1), but we lack evidence on the evolutionary and epidemiological significance of these changes. Conversely, there are many pathogen and host traits of key epidemiological importance that may be epigenetically controlled, some of which may have transgenerational consequences and whose mechanistic basis would merit further investigation.

In conclusion, the future is bright for the epigenetics of host–pathogen interactions. We are confident that in the next few years, cutting-edge epigenomic techniques combined with experimental (whole organism), functional, and theoretical (modeling) approaches will provide fascinating insights into the interrelations between genetic, epigenetic, and phenotypic variation in the complex world of host–pathogen relationships (Figure S2).

## Supporting Information

Figure S1 **Experimental approach to detect transgenerational epigenetic and phenotypic changes of infection in a model study involving mosquitoes.** Starting from isogenic lines and controlled environmental conditions, female mosquitoes are experimentally infected for successive generations to detect adaptive traits in response to a continuous selection pressure (i.e., infection). Phenotype (behavior, immune response, and physiology), epigenotype, and fitness (i.e., fecundity, longevity, and survival) are then quantified and statistically compared. In F1, two groups of females, either infected or noninfected, are back-crossed with noninfected mosquito control males (NI(C)). If the descendants of infected (I×NI(C)) versus noninfected lines (NI(C)×NI) are phenotypically different but show significant divergence in epigenetic profiles, gene or protein expression—in spite of being still identical at the DNA level—this will be evidence for epigenetically based phenotypic change. In subsequent generations, the comparison of infected versus noninfected mosquito groups that descend of infected mosquito females will allow us to test transient versus stable changes (i.e., adaptive traits) as well as cumulative effects of infection (we may expect them to be greater in V than in III). In addition, differences between the descendants of IV–V in Fx will be indicative of maternal effects.
(PDF)

Figure S2 **Workflow on research strategies in host–parasite epigenetics.** First, a phenomic (experimental) approach in laboratory or field settings can be designed to establish transgenerational phenotypic effects and fitness consequences of host or pathogen evolutionary-relevant traits for infection. Second, epigenetic and functional approaches can then be conducted to examine the mechanistic basis, regulatory pathways, and functional significance of these effects. Third, modeling approaches can be used to model the long-term consequences of the observed transgenerational changes, the dynamics and persistence of different types of epigenetic variation, and the interplay between epigenetic and genetic variation. Arrows indicate interrelationships among the different approaches. Feedback among the different stages (dashed arrows) can serve to generate new hypotheses and test model predictions.
(PDF)

Table S1 **Methodologies for epigenetic analyses.**
(DOCX)

## References

[1] Waddington CH (1942) The epigenome. Endevour 1: 18–20.

[2] Jablonka E, Lamb MJ (2002) The changing concept of epigenetics. In: VanSpeybroeck L, VandeVijver G, DeWaele D, editors. From epigenesis to epigenetics: the genome in context. New York: New York Academy of Sciences. pp. 82–96. 10.1111/j.1749-6632.2002.tb04913.x 12547675

[3] Suzuki MM, Bird A (2008) DNA methylation landscapes: provocative insights from epigenomics. Nature Reviews in Genetics 9: 465–476. 10.1038/nrg2341 18463664

[4] Bannister AJ, Kouzarides T (2011) Regulation of chromatin by histone modifications. Cell Research 21: 381–395. 21321607 10.1038/cr.2011.22 PMC3193420

[5] Storz G (2002) An expanding universe of noncoding RNAs. Science 296: 1260–1263. 12016301 10.1126/science.1072249

[6] Moczek AP, Snell-Rood EC (2008) The basis of bee-ing different: the role of gene silencing in plasticity. Evolution & Development 10: 511–513. 18803767 10.1111/j.1525-142X.2008.00264.x

[7] Kucharski R, Maleszka J, Foret S, Maleszka R (2008) Nutritional control of reproductive status in honeybees via DNA methylation. Science 319: 1827–1830. 18339900 10.1126/science.1153069

[8] Jablonka E, Raz G (2009) Transgenerational epigenetic inheritance: prevalence, mechanisms, and implications for the study of heredity and evolution. The Quarterly Review of Biology 84: 131–176. 19606595 10.1086/598822

[9] Bonduriansky R, Day T (2009) Nongenetic inheritance and its evolutionary implications. Annual Review of Ecology, Evolution, and Systematics 40: 103–125.

[10] Cubas P, Vincent C, Coen E (1999) An epigenetic mutation responsible for natural variation in floral symmetry. Nature 401: 157–161. 10490023 10.1038/43657

[11] Esteller M (2007) Cancer epigenomics: DNA methylomes and histone-modification maps. Nature Reviews in Genetics 8: 286–298. 10.1038/nrg2005 17339880

[12] Reece SE, Ricardo SR, Daniel HN (2009) Plastic parasites: sophisticated strategies for survival and reproduction? Evolutionary Applications 2: 11–23. 20305703 10.1111/j.1752-4571.2008.00060.x PMC2836026

[13] Poulin R, Thomas F (2008) Epigenetic effects of infection on the phenotype of host offspring: parasites reaching across host generations. Oikos 117: 331–335.

[14] Rando OJ, Verstrepen KJ (2007) Timescales of genetic and epigenetic inheritance. Cell 128: 655–668. 17320504 10.1016/j.cell.2007.01.023

[15] Merrick CJ, Duraisingh MT (2010) Epigenetics in *Plasmodium*: what do we really know? Eukaryotic Cell 9: 1150–1158. 20562224 10.1128/EC.00093-10 PMC2918939

[16] Meissner M, Soldati D (2005) The transcription machinery and the molecular toolbox to control gene expression in Toxoplasma gondii and other protozoan parasites. Microbes and Infection 7: 1376–1384. 16087378 10.1016/j.micinf.2005.04.019

[17] Hakimi M-A, Deitsch KW (2007) Epigenetics in Apicomplexa: control of gene expression during cell cycle progression, differentiation and antigenic variation. Current Opinion in Microbiology 10: 357–362. 17719264 10.1016/j.mib.2007.07.005

[18] Croken MM, Nardelli SC, Kim K (2012) Chromatin modifications, epigenetics, and how protozoan parasites regulate their lives. Trends in Parasitology 28: 202–213. 22480826 10.1016/j.pt.2012.02.009 PMC3340475

[19] Bougdour A, Maubon D, Baldacci P, Ortet P, Bastien O, et al (2009) Drug inhibition of HDAC3 and epigenetic control of differentiation in Apicomplexa parasites. Journal of Experimental Medicine 206: 953–966. 19349466 10.1084/jem.20082826 PMC2715132

[20] Dixon SE, Stilger KL, Elias EV, Naguleswaran A, Sullivan Jr WJ (2010) A decade of epigenetic research in *Toxoplasma gondii* . Molecular and Biochemical Parasitology 173: 1–9. 20470832 10.1016/j.molbiopara.2010.05.001 PMC2886187

[21] Mishra PK, Baum M, Carbon J (2011) DNA methylation regulates phenotype-dependent transcriptional activity in *Candida albicans* . Proc Natl Acad Sci 108: 11965–11970. 21730141 10.1073/pnas.1109631108 PMC3141964

[22] Chookajorn T, Dzikowski R, Frank M, Li F, Jiwani AZ, et al (2007) Epigenetic memory at malaria virulence genes. Proc Natl Acad Sci 104: 899–902. 17209011 10.1073/pnas.0609084103 PMC1764221

[23] Rovira-Graells N, Gupta AP, Planet E, Crowley VM, Mok S, et al. (in press) Transcriptional variation in the malaria parasite *Plasmodium falciparum*. Genome Research. 10.1101/gr.129692.111 PMC3337437 22415456

[24] Volz JC, Bártfai R, Petter M, Langer C, Josling GA, et al (2012) PfSET10, a *Plasmodium falciparum* methyltransferase, maintains the active *var* gene in a poised state during parasite division. Cell Host & Microbe 11: 7–18. 22264509 10.1016/j.chom.2011.11.011

[25] Huguenin M, Bracha R, Chookajorn T, Mirelman D (2010) Epigenetic transcriptional gene silencing in *Entamoeba histolytica*: insight into histone and chromatin modifications. Parasitology 137: 619–627. 19849886 10.1017/S0031182009991363

[26] Løbner-Olesen A, Skovgaard O, Marinus MG (2005) Dam methylation: coordinating cellular processes. Current Opinion in Microbiology 8: 154–160. 15802246 10.1016/j.mib.2005.02.009

[27] Heithoff DM, Sinsheimer RL, Low DA, Mahan MJ (1999) An essential role for DNA adenine methylation in bacterial virulence. Science 284: 967–970. 10320378 10.1126/science.284.5416.967

[28] Marinus MG, Casadesus J (2009) Roles of DNA adenine methylation in host–pathogen interactions: mismatch repair, transcriptional regulation, and more. FEMS Microbiology Reviews 33: 488–503. 19175412 10.1111/j.1574-6976.2008.00159.x PMC2941194

[29] Handel A, Ebers G, Ramagopalan S (2010) Epigenetics: molecular mechanisms and implications for disease. Trends in Molecular Medicine 16: 7–23. 20022812 10.1016/j.molmed.2009.11.003

[30] Lefèvre T, Lebarbenchon C, Gauthier-Clerc M, Missé D, Poulin R, et al (2009) The ecological significance of manipulative parasites. Trends in Ecology & Evolution 24: 41–48. 19026461 10.1016/j.tree.2008.08.007

[31] Hurd H (2009) Chapter 4 evolutionary drivers of parasite-induced changes in insect life-history traits: from theory to underlying mechanisms. In: Joanne PW, editor. Advances in parasitology. Academic Press. pp. 85–110. 10.1016/S0065-308X(08)00604-0 19289191

[32] Bhavsar A, Guttman J, Finlay B (2007) Manipulation of host-cell pathways by bacterial pathogens. Nature 449: 827–861. 17943119 10.1038/nature06247

[33] Hamon MA, Cossart P (2008) Histone modifications and chromatin remodeling during bacterial infections. Cell Host & Microbe 4: 100–109. 18692770 10.1016/j.chom.2008.07.009

[34] Paschos K, Allday MJ (2010) Epigenetic reprogramming of host genes in viral and microbial pathogenesis. Trends in Microbiology 18: 439–447. 20724161 10.1016/j.tim.2010.07.003 PMC3089700

[35] Ribet D, Cossart P (2010) Post-translational modifications in host cells during bacterial infection. FEBS Letters 584: 2748–2806. 20493189 10.1016/j.febslet.2010.05.012

[36] Pennini ME, Pai RK, Schultz DC, Boom WH, Harding CV (2006) *Mycobacterium tuberculosis* 19-kDa lipoprotein inhibits IFN-γ-induced chromatin remodeling of MHC2TA by TLR2 and MAPK signaling. The Journal of Immunology 176: 4323–4330. 16547269 10.4049/jimmunol.176.7.4323

[37] Marazzi I, Ho JSY, Kim J, Manicassamy B, Dewell S, et al (2012) Suppression of the antiviral response by an influenza histone mimic. Nature 483: 428–433. 22419161 10.1038/nature10892 PMC3598589

[38] Werren JH, Baldo L, Clark ME (2008) *Wolbachia*: master manipulators of invertebrate biology. Nature Review in Microbiology 6: 741–751. 18794912 10.1038/nrmicro1969

[39] Negri I, Franchini A, Gonella E, Daffonchio D, Mazzoglio PJ, et al (2009) Unravelling the *Wolbachia* evolutionary role: the reprogramming of the host genomic imprinting. Proc Biol Sci 276(1666): 2485–2491. 19364731 10.1098/rspb.2009.0324 PMC2690474

[40] Saridaki A, Sapountzis P, Harris HL, Batista PD, Biliske JA, et al (2011) *Wolbachia* prophage DNA adenine methyltransferase genes in different *Drosophila-Wolbachia* associations. PLoS ONE 6: e19708 doi:10.1371/journal.pone.0019708. 21573076 10.1371/journal.pone.0019708 PMC3089641

[41] Youngblood B, Davis CW, Ahmed R (2010) Making memories that last a lifetime: heritable functions of self-renewing memory CD8 T cells. International Immunology 22: 797–803. 20732857 10.1093/intimm/dxq437 PMC2946216

[42] Conrath U (2011) Molecular aspects of defence priming. Trends in Plant Science 16: 524–531. 21782492 10.1016/j.tplants.2011.06.004

[43] Boyko A, Kovalchuk I (2011) Genetic and epigenetic effects of plant–pathogen interactions: an evolutionary perspective. Molecular Plant 4: 1014–1023. 21459830 10.1093/mp/ssr022

[44] Luna E, Bruce TJA, Roberts MR, Flors V, Ton J (2012) Next-generation systemic acquired resistance. Plant Physiology 158: 844–853. 22147520 10.1104/pp.111.187468 PMC3271772

[45] Relton CL, Davey Smith G (2010) Epigenetic epidemiology of common complex disease: prospects for prediction, prevention, and treatment. PLoS Med 7: e1000356 doi:10.1371/journal.pmed.1000356. 21048988 10.1371/journal.pmed.1000356 PMC2964338

[46] Relton CL, Davey Smith G (2012) Two-step epigenetic Mendelian randomization: a strategy for establishing the causal role of epigenetic processes in pathways to disease. International Journal of Epidemiology 41: 161–176. 22422451 10.1093/ije/dyr233 PMC3304531

[47] Fraga MF, Esteller M (2007) Epigenetics and aging: the targets and the marks. Trends in Genetics 23: 413–418. 17559965 10.1016/j.tig.2007.05.008

[48] Hemberger M, Dean W, Reik W (2009) Epigenetic dynamics of stem cells and cell lineage commitment: digging Waddington's canal. Nature Reviews in Molecular Cell Biology 10: 526–537. 19603040 10.1038/nrm2727

[49] Ferguson-Smith AC, Surani MA (2001) Imprinting and the epigenetic asymmetry between parental genomes. Science 293: 1086–1089. 11498578 10.1126/science.1064020

[50] Relton CL, Davey Smith G (2012) Is epidemiology ready for epigenetics? International Journal of Epidemiology 41: 5–9. 22422447 10.1093/ije/dys006 PMC3304535

[51] Baccarelli A, Ghosh S (2012) Environmental exposures, epigenetics and cardiovascular disease. Current Opinion in Clinical Nutrition & Metabolic Care 15: 323–329. 22669047 10.1097/MCO.0b013e328354bf5c PMC3742092

[52] Richards E (2008) Population epigenetics. Current Opinion in Genetics & Development 18: 221–227. 18337082 10.1016/j.gde.2008.01.014

[53] Carius HJ, Little TJ, Ebert D (2001) Genetic variation in a host-parasite association: potential for coevolution and frequency-dependent selection. Evolution 55: 1136–1145. 11475049 10.1111/j.0014-3820.2001.tb00633.x

[54] Lambrechts L, Fellous S, Koella JC (2006) Coevolutionary interactions between host and parasite genotypes. Trends in Parasitology 22: 12–16. 16310412 10.1016/j.pt.2005.11.008

[55] Stjernman M, Little TJ (2011) Genetic variation for maternal effects on parasite susceptibility. Journal of Evolutionary Biology 24: 2357–2363. 21848987 10.1111/j.1420-9101.2011.02363.x

[56] Cosseau C, Azzi A, Rognon A, Boissier J, Gourbière S, et al (2010) Epigenetic and phenotypic variability in populations of *Schistosoma mansoni*—a possible kick-off for adaptive host/parasite evolution. Oikos 119: 669–678.

[57] Ebert D (2008) Host–parasite coevolution: insights from the daphnia–parasite model system. Current Opinion in Microbiology 11: 290–301. 18556238 10.1016/j.mib.2008.05.012

[58] Vale PF, Wilson AJ, Best A, Boots M, Little TJ (2011) Epidemiological, evolutionary, and coevolutionary implications of context-dependent parasitism. The American Naturalist 177: 510–521. 10.1086/659002 PMC3725425 21460572

[59] Feil R, Fraga MF (2012) Epigenetics and the environment: emerging patterns and implications. Nature Reviews in Genetics 13: 97–109. 10.1038/nrg3142 22215131

[60] Laine A-L (2008) Temperature-mediated patterns of local adaptation in a natural plant–pathogen metapopulation. Ecology Letters 11: 327–337. 18248450 10.1111/j.1461-0248.2007.01146.x

[61] Wolinska J, King KC (2009) Environment can alter selection in host–parasite interactions. Trends in Parasitology 25: 236–244. 19356982 10.1016/j.pt.2009.02.004

[62] Moret Y, Schmid-Hempel P (2001) Entomology—immune defence in bumble-bee offspring. Nature 414: 506–506. 10.1038/35107138 11734840

[63] Little TJ, O'Connor B, Colegrave N, Watt K, Read AF (2003) Maternal transfer of strain-specific immunity in an invertebrate. Current Biology 13: 489–492. 12646131 10.1016/s0960-9822(03)00163-5

[64] Kurtz J, Franz K (2003) Innate defence: evidence for memory in invertebrate immunity. Nature 425: 37–38. 12955131 10.1038/425037a

[65] Bossdorf O, Richards CL, Pigliucci M (2008) Epigenetics for ecologists. Ecology Letters 11: 106–115. 18021243 10.1111/j.1461-0248.2007.01130.x

[66] Ho D, Burggren W (2010) Epigenetics and transgenerational transfer: a physiological perspective. The Journal of Experimental Biology 213: 3–19. 20008356 10.1242/jeb.019752

[67] Hunter B, Hollister J, Bomblies K (2012) Epigenetic inheritance: what news for evolution? Current Biology 22: R54–R56. 22280908 10.1016/j.cub.2011.11.054

[68] Kouzarides T (2007) Chromatin modifications and their function. Cell 128: 693–705. 17320507 10.1016/j.cell.2007.02.005

[69] Jenuwein T, Allis CD (2001) Translating the histone code. Science 293: 1074–1080. 11498575 10.1126/science.1063127

[70] Wion D, Casadesus J (2006) N6-methyl-adenine: an epigenetic signal for DNA-protein interactions. Nature Reviews in Microbiology 4: 183–192. 16489347 10.1038/nrmicro1350 PMC2755769

[71] Jeltsch A (2010) Phylogeny of methylomes. Science 328: 837–838. 20466912 10.1126/science.1190738

[72] Zemach A, McDaniel IE, Silva P, Zilberman D (2010) Genome-wide evolutionary analysis of eukaryotic DNA methylation. Science 328: 916–919. 20395474 10.1126/science.1186366

[73] Guil S, Esteller M (2009) DNA methylomes, histone codes and miRNAs: tying it all together. The International Journal of Biochemistry & Cell Biology 41: 87–95. 18834952 10.1016/j.biocel.2008.09.005

[74] Laird PW (2010) Principles and challenges of genomewide DNA methylation analysis. Nature Reviews in Genetics 11: 191–203. 10.1038/nrg2732 20125086

[75] Eick D, Fritz HJ, Doerfler W (1983) Quantitative determination of 5-methylcytosine in DNA by reverse-phase high-performance liquid chromatography. Analytical Biochemistry 135: 165–171. 6422793 10.1016/0003-2697(83)90746-7

[76] Fraga MF, Rodriguez R, Canal MJ (2000) Rapid quantification of DNA methylation by high performance capillary electrophoresis. Electrophoresis 21: 2990–2994. 11001314 10.1002/1522-2683(20000801)21:14<2990::AID-ELPS2990>3.0.CO;2-I

[77] Karimi M, Johansson S, Stach D, Corcoran M, Grander D, et al (2006) LUMA (LUminometric Methylation Assay)–a high throughput method to the analysis of genomic DNA methylation. Experimental Cell Research 312: 1989–1995. 16624287 10.1016/j.yexcr.2006.03.006

[78] Ronaghi M, Uhlen M, Nyren P (1998) A sequencing method based on real-time pyrophosphate. Science 281: 363, 365. 10.1126/science.281.5375.363 9705713

[79] Clark SJ, Harrison J, Paul CL, Frommer M (1994) High sensitivity mapping of methylated cytosines. Nucleic Acids Research 22: 2990–2997. 8065911 10.1093/nar/22.15.2990 PMC310266

[80] Jordà M, Rodríguez J, Frigola J, Peinado MA (2009) Analysis of DNA Methylation by Amplification of Intermethylated Sites (AIMS). In: Tost J, editor. DNA methylation: methods and protocols, second edition. Humana Press. pp. 107–116.

[81] Frigola J, Sole X, Paz MF, Moreno V, Esteller M, et al (2005) Differential DNA hypermethylation and hypomethylation signatures in colorectal cancer. Human Molecular Genetics 14: 319–326. 15574462 10.1093/hmg/ddi028

[82] Wang Y, Jorda M, Jones PL, Maleszka R, Ling X, et al (2006) Functional CpG methylation system in a social insect. Science 314: 645–647. 17068262 10.1126/science.1135213

[83] Weber M, Davies JJ, Wittig D, Oakeley EJ, Haase M, et al (2005) Chromosome-wide and promoter-specific analyses identify sites of differential DNA methylation in normal and transformed human cells. Nature Genetics 37: 853–862. 16007088 10.1038/ng1598

[84] Down TA, Rakyan VK, Turner DJ, Flicek P, Li H, et al (2008) A Bayesian deconvolution strategy for immunoprecipitation-based DNA methylome analysis. Nature Biotechnology 26: 779–785. 10.1038/nbt1414 PMC2644410 18612301

[85] Lister R, Pelizzola M, Dowen RH, Hawkins RD, Hon G, et al (2009) Human DNA methylomes at base resolution show widespread epigenomic differences. Nature 462: 315–322. 19829295 10.1038/nature08514 PMC2857523

[86] Clarke J, Wu HC, Jayasinghe L, Patel A, Reid S, et al (2009) Continuous base identification for single-molecule nanopore DNA sequencing. Nature Nanotechnology 4: 265–270. 10.1038/nnano.2009.12 19350039

[87] Flusberg BA, Webster DR, Lee JH, Travers KJ, Olivares EC, et al (2010) Direct detection of DNA methylation during single-molecule, real-time sequencing. Nature Methods 7: 461–465. 20453866 10.1038/nmeth.1459 PMC2879396

[88] Fraga MF, Ballestar E, Villar-Garea A, Boix-Chornet M, Espada J, et al (2005) Loss of acetylation at Lys16 and trimethylation at Lys20 of histone H4 is a common hallmark of human cancer. Nature Genetics 37: 391–400. 15765097 10.1038/ng1531

[89] Carey MF, Peterson CL, Smale ST (2009) Chromatin immunoprecipitation (ChIP). Cold Spring Harbor Protocols 2009: pdb prot5279. 10.1101/pdb.prot5279 20147264

[90] Solomon MJ, Larsen PL, Varshavsky A (1988) Mapping protein-DNA interactions in vivo with formaldehyde: evidence that histone H4 is retained on a highly transcribed gene. Cell 53: 937–947. 2454748 10.1016/s0092-8674(88)90469-2

[91] Kurdistani SK, Tavazoie S, Grunstein M (2004) Mapping global histone acetylation patterns to gene expression. Cell 117: 721–733. 15186774 10.1016/j.cell.2004.05.023

[92] Barski A, Cuddapah S, Cui K, Roh TY, Schones DE, et al (2007) High-resolution profiling of histone methylations in the human genome. Cell 129: 823–837. 17512414 10.1016/j.cell.2007.05.009

[93] Cui L, Miao J (2010) Chromatin-mediated epigenetic regulation in the malaria parasite *Plasmodium falciparum* . Eukaryotic Cell 9: 1138–1149. 20453074 10.1128/EC.00036-10 PMC2918932

[94] Chookajorn T, Ponsuwanna P, Cui L (2008) Mutually exclusive var gene expression in the malaria parasite: multiple layers of regulation. Trends in Parasitology 24: 455–461. 18771955 10.1016/j.pt.2008.07.005

[95] Sonda S, Morf L, Bottova I, Baetschmann H, Rehrauer H, et al (2010) Epigenetic mechanisms regulate stage differentiation in the minimized protozoan *Giardia lamblia* . Molecular Microbiology 76: 48–67. 20132448 10.1111/j.1365-2958.2010.07062.x

[96] Geyer KK, Rodriguez Lopez CM, Chalmers IW, Munshi SE, Truscott M, et al (2011) Cytosine methylation regulates oviposition in the pathogenic blood fluke *Schistosoma mansoni* . Nature Communications 2: 424. 10.1038/ncomms1433 PMC3265374 21829186

[97] Fernandez AF, Rosales C, Lopez-Nieva P, Graña O, Ballestar E, et al (2009) The dynamic DNA methylomes of double-stranded DNA viruses associated with human cancer. Genome Research 19: 438–451. 19208682 10.1101/gr.083550.108 PMC2661803

[98] Fonseca GJ, Thillainadesan G, Yousef AF, Ablack JN, Mossman KL, et al (2012) Adenovirus evasion of interferon-mediated innate immunity by direct antagonism of a cellular histone posttranslational modification. Cell Host & Microbe 11: 597–606. 22704620 10.1016/j.chom.2012.05.005

[99] Lang C, Hildebrandt A, Brand F, Opitz L, Dihazi H, et al (2012) Impaired chromatin remodelling at STAT1-regulated promoters leads to global unresponsiveness of *Toxoplasma gondii* infected macrophages to IFN-γ. PLoS Pathog 8: e1002483 doi:10.1371/journal.ppat.1002483. 22275866 10.1371/journal.ppat.1002483 PMC3262016

[100] Garcia-Garcia JC, Barat NC, Trembley SJ, Dumler JS (2009) Epigenetic silencing of host cell defense genes enhances intracellular survival of the rickettsial pathogen *Anaplasma phagocytophilum* . PLoS Pathog 5: e1000488 doi:10.1371/journal.ppat.1000488. 19543390 10.1371/journal.ppat.1000488 PMC2694362

