# Supplementary figures and images for: Epigenetics of Host–Pathogen Interactions: The Road Ahead and the Road Behind
Source: PLoS Pathog. 2012 Nov 29;8(11):e1003007. doi: 10.1371/journal.ppat.1003007 (PMC3510240; doi:10.1371/journal.ppat.1003007)

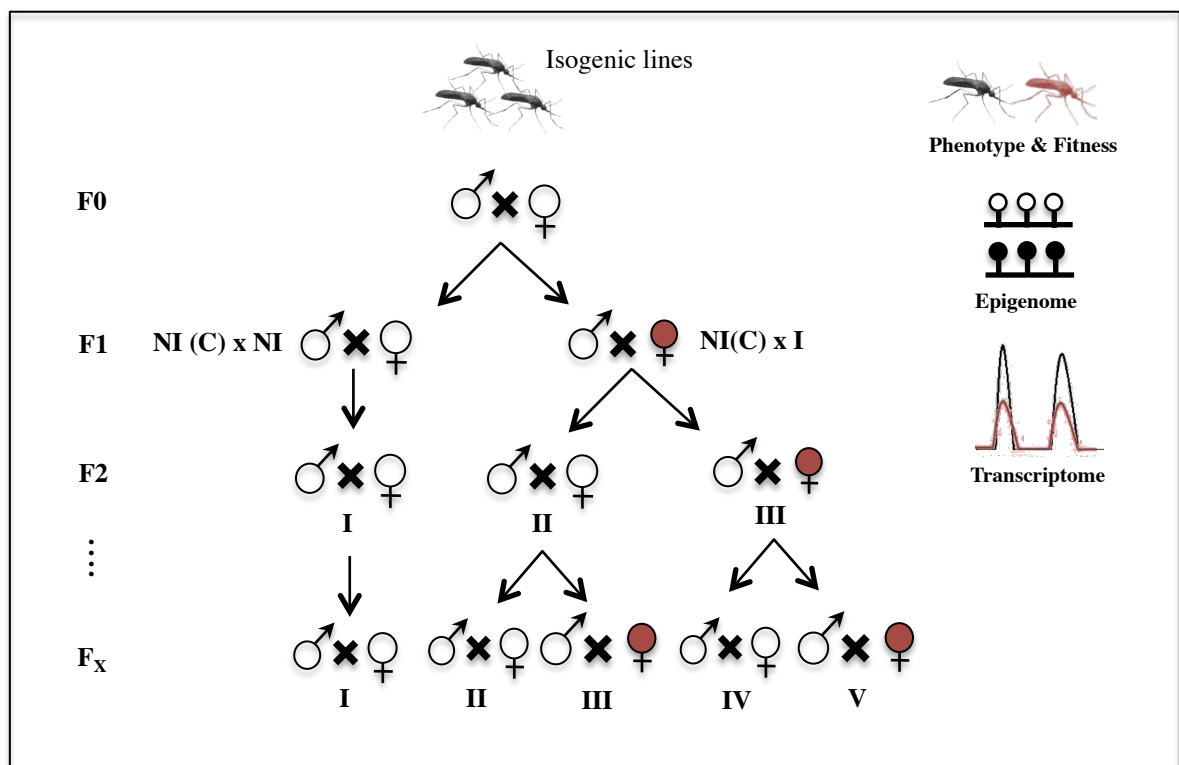

Supplement: Figure S1 — Experimental approach to detect transgenerational epigenetic and phenotypic changes of infection in a model study involving mosquitoes. Starting from isogenic lines and controlled environmental conditions, female mosquitoes are experimentally infected for successive generations to detect adaptive traits in response to a continuous selection pressure (i.e., infection). Phenotype (behavior, immune response, and physiology), epigenotype, and fitness (i.e., fecundity, longevity, and survival) are then quantified and statistically compared. In F1, two groups of females, either infected or noninfected, are back-crossed with noninfected mosquito control males (NI(C)). If the descendants of infected (I×NI(C)) versus noninfected lines (NI(C)×NI) are phenotypically different but show significant divergence in epigenetic profiles, gene or protein expression—in spite of being still identical at the DNA level—this will be evidence for epigenetically based phenotypic change. In subsequent generations, the comparison of infected versus noninfected mosquito groups that descend of infected mosquito females will allow us to test transient versus stable changes (i.e., adaptive traits) as well as cumulative effects of infection (we may expect them to be greater in V than in III). In addition, differences between the descendants of IV–V in Fx will be indicative of maternal effects. (PDF) [file ppat.1003007.s001.pdf]

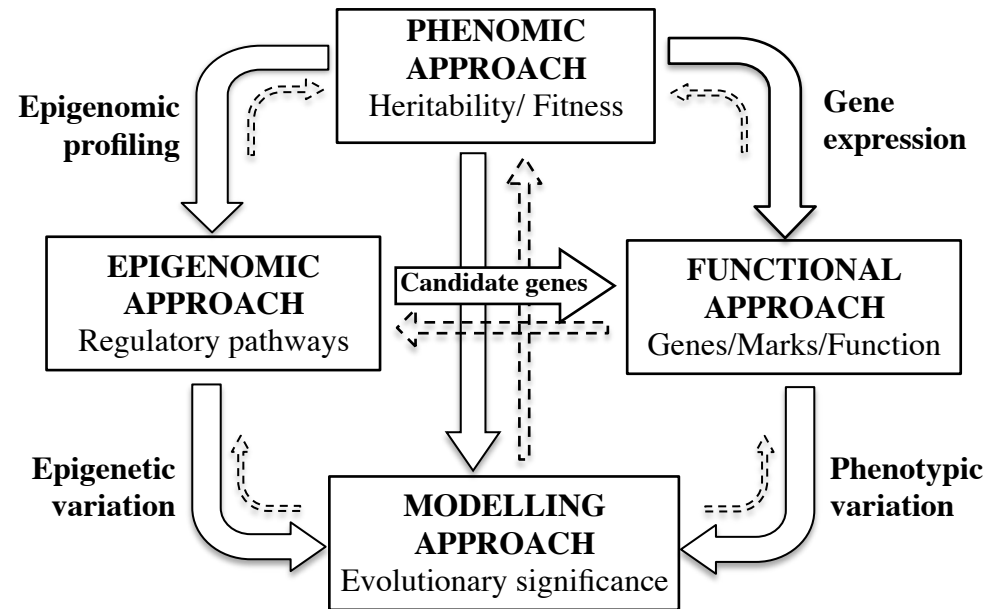

Supplement: Figure S2 — Workflow on research strategies in host–parasite epigenetics. First, a phenomic (experimental) approach in laboratory or field settings can be designed to establish transgenerational phenotypic effects and fitness consequences of host or pathogen evolutionary-relevant traits for infection. Second, epigenetic and functional approaches can then be conducted to examine the mechanistic basis, regulatory pathways, and functional significance of these effects. Third, modeling approaches can be used to model the long-term consequences of the observed transgenerational changes, the dynamics and persistence of different types of epigenetic variation, and the interplay between epigenetic and genetic variation. Arrows indicate interrelationships among the different approaches. Feedback among the different stages (dashed arrows) can serve to generate new hypotheses and test model predictions. (PDF) [file ppat.1003007.s002.pdf]
