# Supplementary material for: Epigenetics of Host–Pathogen Interactions: The Road Ahead and the Road Behind
Source: PLoS Pathog. 2012 Nov 29;8(11):e1003007. doi: 10.1371/journal.ppat.1003007 (PMC3510240; doi:10.1371/journal.ppat.1003007)
Supplement: Table S1 — Methodologies for epigenetic analyses. (DOCX) [file ppat.1003007.s003.docx]

**Table S1. Methodologies for epigenetic analyses^1^**

|  | **Method** | **Discrimination principle** | **Detection technique** | **Resolution** | **References** |
| --- | --- | --- | --- | --- | --- |
| ***DNA Methylation*** | ***Quantification of global 5-methylcytosines*** | | | | |
|  | HPLC, HPCE | Chromatography | Absorbance | 5-mC genomic content | [[1](#_ENREF_1),[2](#_ENREF_2)] |
|  | HPLC/EI-MS | Chromatography | EI-MS | 5-mC genomic content | [[3](#_ENREF_3)] |
|  | LUMA | Methylation-sensitive RE | Pyrosequencing | RE recognition sites within the genome | [[4](#_ENREF_4)] |
|  | ***Sequence specific analyses*** | | | | |
|  | Southern blot | Methylation-sensitive RE | Hybridization | Single RE recognition site | [[5](#_ENREF_5)] |
|  | PCR amplification | Methylation-sensitive RE | PCR | Single RE recognition site | [[6](#_ENREF_6)] |
|  | Bisulphite sequencing | Bisulphite conversion | Sanger sequencing | Single nucleotide within the amplicon | [[7](#_ENREF_7),[8](#_ENREF_8)] |
|  | PyroMeth | Bisulphite conversion | Pyrosequencing | Single nucleotide within the amplicon | [[9](#_ENREF_9)] |
|  | MSP | Bisulphite conversion | PCR | Few CpGs inside the primers | [[10](#_ENREF_10)] |
|  | MethyLight | Bisulphite conversion | Fluorescence real-time PCR | Few CpGs inside the primers | [[11](#_ENREF_11)] |
|  | Headloop-PCR | Bisulphite conversion | PCR | Few CpGs inside the primers | [[12](#_ENREF_12)] |
|  | In-tube melting curves analysis | Bisulphite conversion | Fluorescence real-time PCR | Average methylation of amplicon | [[13](#_ENREF_13)] |
|  | COBRA | Bisulphite conversion + Methylation-sensitive RE | PCR | Single RE recognition site within the amplicon | [[14](#_ENREF_14)] |
|  | Bisulphite-Sequenom | Bisulphite conversion | MALDITOF-MS | Single nucleotide within the amplicon | [[15](#_ENREF_15)] |
|  | MS-SnuPE | Bisulphite conversion | SnuPE | Few CpGs within the amplicon | [[16](#_ENREF_16)] |
|  | MS-SSCA | Bisulphite conversion | SSCA | Relative average methylation of amplicon | [[17](#_ENREF_17)] |
|  | ***Genome-wide analyses*** | | | | |
|  | RLGS | Methylation-sensitive RE | 2D-electrophoresis | RE recognition sites within the genome | [[18](#_ENREF_18)] |
|  | AP-MS-PCR | Methylation sensitive RE | Southern blot | RE recognition sites within the genome | [[19](#_ENREF_19)] |
|  | MS-AFLP | Methylation sensitive RE | Fingerprinting | RE recognition sites within the genome | [[20](#_ENREF_20)] |
|  | MCA | Methylation sensitive RE | RDA | RE recognition sites within the genome | [[21](#_ENREF_21)] |
|  | AIMS | Methylation sensitive RE | Fingerprinting | RE recognition sites within the genome | [[22](#_ENREF_22)] |
|  | MeDIP-chip | 5mC antibody | Microarray | Relative average methylation of the immunoprecipitated sequences that hybridize to microarrayed probes | [[23](#_ENREF_23)] |
|  | MeDIP-seq | 5mC antibody | NGS | Relative average methylation of the immunoprecipitated sequences | [[24](#_ENREF_24)] |
|  | Bisulphite-chip | Bisulphite conversion | Microarray | Few CpGs within the probes of the microarray | [[25](#_ENREF_25)] |
|  | Bisulphite-seq | Bisulphite conversion | NGS | Single nucleotide within the genome | [[26](#_ENREF_26)] |
|  | Nanopore sequencing | Conductivity changes in grapheme nanopores when DNA strands pass through | NGS | Single nucleotide within the genome | [[27](#_ENREF_27)] |
|  | SMRT | SMRT | NGS | Single nucleotide within the genome | [[28](#_ENREF_28)] |

| ***Histone modifications***  ***and variants*** | ***Quantification of histone modifications/variants*** | | | |  |
| --- | --- | --- | --- | --- | --- |
|  | Mass spectrometry | Mass spectrometry | Mass spectrometry | Global content of a specific histone mark/variant | [[29](#_ENREF_29)] |
|  | Western Blot | Antibody | Western Blot | Global content of a specific histone mark/variant | [[30](#_ENREF_30)] |
|  | ***Sequence specific analyses*** | | | | |
|  | ChIP-PCR | Antibody | Conventional PCR or fluorescence real-time PCR | Relative average of the histone mark/variant content within the amplicon | [[31](#_ENREF_31)] |
|  | ***Genome-wide analyses*** | | | | |
|  | ChIP-on-chip | Antibody | Microarray | Relative average of the histone mark/variant content of the immunoprecipitated sequences that hybridize on the microarray | [[32](#_ENREF_32)] |
|  | ChIP-seq | Antibody | NGS | Relative average of the histone mark/variant content of the immunoprecipitated sequences | [[33](#_ENREF_33)] |
| ***Both^2^*** | ChIP-BS-seq | Antibody + Bisulphite conversion | NGS | Single nucleotide within the immunoprecipitated sequences | [[34](#_ENREF_34),[35](#_ENREF_35)] |

^1^ Abbreviations: HPLC, High Performance Liquid Chromatography; EI, Electrospray Ionization; MS, Mass Spectrometry; HPCE, High Performance Capillary Electrophoresis; LUMA, LUminometric Methylation Assay; RE, restriction enzyme; 5mC, 5-methylcytosine; PCR, Polymerase Chain Reaction; MSP: Methylation Specific PCR; COBRA, COmbined Bisulphite Restriction Analysis; MS-SnuPE, Methylation Sensitive-Single nucleotide Primer Extension; MS-SSCA, Methylation Sensitive-Single Conformational Analysis; RLGS, Restriction Landmark Genomic Scanning; AP-MS-PCR, Arbitrarily Primed Methylation Sensitive-PCR; MS-AFLP, Methylation-Sensitive Amplified Fragment Length Polymorphism; MCA, Methylated CpG island Amplification; AIMS, Amplification of Inter-Methylated Sites; chip, microarray; MeDIP, Methyl-DNA Immunoprecipitation; RDA, Representational Difference Analysis; SPM, Segregation of Partially Melted molecules; NGS, Next Generation Sequencing; SMRT, Single Molecule Real Time; ChIP: Chromatin ImmunoPrecipitation.

^2^ DNA methylation + Histone modifications and variants

1. Eick D, Fritz HJ, Doerfler W (1983) Quantitative determination of 5-methylcytosine in DNA by reverse-phase high-performance liquid chromatography. Anal Biochem 135: 165-171.

2. Fraga MF, Rodriguez R, Canal MJ (2000) Rapid quantification of DNA methylation by high performance capillary electrophoresis. Electrophoresis 21: 2990-2994.

3. Friso S, Choi SW, Dolnikowski GG, Selhub J (2002) A method to assess genomic DNA methylation using high-performance liquid chromatography/electrospray ionization mass spectrometry. Anal Chem 74: 4526-4531.

4. Karimi M, Johansson S, Stach D, Corcoran M, Grander D, et al. (2006) LUMA (LUminometric Methylation Assay)--a high throughput method to the analysis of genomic DNA methylation. Exp Cell Res 312: 1989-1995.

5. Gounari F, Banks GR, Khazaie K, Jeggo PA, Holliday R (1987) Gene reactivation: a tool for the isolation of mammalian DNA methylation mutants. Genes Dev 1: 899-912.

6. Kutueva LI, Ashapkin VV, Vanyushin BF (1996) The methylation pattern of a cytosine DNA-methyltransferase gene in Arabidopsis thaliana plants. Biochem Mol Biol Int 40: 347-353.

7. Frommer M, McDonald LE, Millar DS, Collis CM, Watt F, et al. (1992) A genomic sequencing protocol that yields a positive display of 5-methylcytosine residues in individual DNA strands. Proc Natl Acad Sci U S A 89: 1827-1831.

8. Clark SJ, Harrison J, Paul CL, Frommer M (1994) High sensitivity mapping of methylated cytosines. Nucleic Acids Res 22: 2990-2997.

9. Uhlmann K, Brinckmann A, Toliat MR, Ritter H, Nurnberg P (2002) Evaluation of a potential epigenetic biomarker by quantitative methyl-single nucleotide polymorphism analysis. Electrophoresis 23: 4072-4079.

10. Herman JG, Graff JR, Myohanen S, Nelkin BD, Baylin SB (1996) Methylation-specific PCR: a novel PCR assay for methylation status of CpG islands. Proc Natl Acad Sci U S A 93: 9821-9826.

11. Eads CA, Danenberg KD, Kawakami K, Saltz LB, Blake C, et al. (2000) MethyLight: a high-throughput assay to measure DNA methylation. Nucleic Acids Res 28: E32.

12. Rand KN, Ho T, Qu W, Mitchell SM, White R, et al. (2005) Headloop suppression PCR and its application to selective amplification of methylated DNA sequences. Nucleic Acids Res 33: e127.

13. Worm J, Aggerholm A, Guldberg P (2001) In-tube DNA methylation profiling by fluorescence melting curve analysis. Clin Chem 47: 1183-1189.

14. Xiong Z, Laird PW (1997) COBRA: a sensitive and quantitative DNA methylation assay. Nucleic Acids Res 25: 2532-2534.

15. Ehrich M, Nelson MR, Stanssens P, Zabeau M, Liloglou T, et al. (2005) Quantitative high-throughput analysis of DNA methylation patterns by base-specific cleavage and mass spectrometry. Proc Natl Acad Sci U S A 102: 15785-15790.

16. Gonzalgo ML, Jones PA (2002) Quantitative methylation analysis using methylation-sensitive single-nucleotide primer extension (Ms-SNuPE). Methods 27: 128-133.

17. Bianco T, Hussey D, Dobrovic A (1999) Methylation-sensitive, single-strand conformation analysis (MS-SSCA): A rapid method to screen for and analyze methylation. Hum Mutat 14: 289-293.

18. Hatada I, Hayashizaki Y, Hirotsune S, Komatsubara H, Mukai T (1991) A genomic scanning method for higher organisms using restriction sites as landmarks. Proc Natl Acad Sci U S A 88: 9523-9527.

19. Gonzalgo ML, Liang G, Spruck CH, 3rd, Zingg JM, Rideout WM, 3rd, et al. (1997) Identification and characterization of differentially methylated regions of genomic DNA by methylation-sensitive arbitrarily primed PCR. Cancer Res 57: 594-599.

20. Yamamoto F, Yamamoto M, Soto JL, Kojima E, Wang EN, et al. (2001) Notl-Msell methylation-sensitive amplied fragment length polymorhism for DNA methylation analysis of human cancers. Electrophoresis 22: 1946-1956.

21. Toyota M, Ho C, Ahuja N, Jair KW, Li Q, et al. (1999) Identification of differentially methylated sequences in colorectal cancer by methylated CpG island amplification. Cancer Res 59: 2307-2312.

22. Frigola J, Ribas M, Risques RA, Peinado MA (2002) Methylome profiling of cancer cells by amplification of inter-methylated sites (AIMS). Nucleic Acids Res 30: e28.

23. Weber M, Davies JJ, Wittig D, Oakeley EJ, Haase M, et al. (2005) Chromosome-wide and promoter-specific analyses identify sites of differential DNA methylation in normal and transformed human cells. Nat Genet 37: 853-862.

24. Down TA, Rakyan VK, Turner DJ, Flicek P, Li H, et al. (2008) A Bayesian deconvolution strategy for immunoprecipitation-based DNA methylome analysis. Nat Biotechnol 26: 779-785.

25. Bibikova M, Le J, Barnes B, Saedinia-Melnyk S, Zhou L, et al. (2009) Genome-wide DNA methylation profiling using Infinium(R) assay. Epigenomics 1: 177-200.

26. Lister R, Pelizzola M, Dowen RH, Hawkins RD, Hon G, et al. (2009) Human DNA methylomes at base resolution show widespread epigenomic differences. Nature.

27. Clarke J, Wu HC, Jayasinghe L, Patel A, Reid S, et al. (2009) Continuous base identification for single-molecule nanopore DNA sequencing. Nat Nanotechnol 4: 265-270.

28. Flusberg BA, Webster DR, Lee JH, Travers KJ, Olivares EC, et al. (2010) Direct detection of DNA methylation during single-molecule, real-time sequencing. Nat Methods 7: 461-465.

29. Fraga MF, Ballestar E, Villar-Garea A, Boix-Chornet M, Espada J, et al. (2005) Loss of acetylation at Lys16 and trimethylation at Lys20 of histone H4 is a common hallmark of human cancer. Nat Genet 37: 391-400.

30. Egelhofer TA, Minoda A, Klugman S, Lee K, Kolasinska-Zwierz P, et al. (2011) An assessment of histone-modification antibody quality. Nat Struct Mol Biol 18: 91-93.

31. Solomon MJ, Larsen PL, Varshavsky A (1988) Mapping protein-DNA interactions in vivo with formaldehyde: evidence that histone H4 is retained on a highly transcribed gene. Cell 53: 937-947.

32. Kurdistani SK, Tavazoie S, Grunstein M (2004) Mapping global histone acetylation patterns to gene expression. Cell 117: 721-733.

33. Barski A, Cuddapah S, Cui K, Roh TY, Schones DE, et al. (2007) High-resolution profiling of histone methylations in the human genome. Cell 129: 823-837.

34. Statham AL, Robinson MD, Song JZ, Coolen MW, Stirzaker C, et al. (2012) Bisulphite-sequencing of chromatin immunoprecipitated DNA (BisChIP-seq) directly informs methylation status of histone-modified DNA. Genome Res.

35. Brinkman AB, Gu H, Bartels SJ, Zhang Y, Matarese F, et al. (2012) Sequential ChIP-bisulfite sequencing enables direct genome-scale investigation of chromatin and DNA methylation cross-talk. Genome Res.
